# Early pain in females is linked to late pathological features in murine experimental osteoarthritis

**DOI:** 10.7717/peerj.15482

**Published:** 2023-06-22

**Authors:** Natália Valdrighi, Arjen B. Blom, Henk M. van Beuningen, Elly L. Vitters, Monique M. Helsen, Birgitte Walgreen, Peter L.E.M. van Lent, Marije I. Koenders, Peter M. van der Kraan, Fons A.J. van de Loo, Esmeralda N. Blaney Davidson

**Affiliations:** Experimental Rheumatology, Radboud University Medical Center, Nijmegen, Netherlands

**Keywords:** Osteoarthritis, Pain, Sex differences, Synovitis

## Abstract

**Background:**

Osteoarthritis (OA) is a progressive joint disease and a major cause of chronic pain in adults. The prevalence of OA is higher in female patients, who tend to have worse OA outcomes, partially due to pain. The association between joint pain and OA pathology is often inconclusive. Preclinical research studies have largely overlooked sex as a potential determinant in joint pain during OA. This study aimed to investigate the role of sex in joint pain in the collagenase-induced OA (CiOA) model and its link with joint pathology.

**Methods:**

Multiple aspects of pain were evaluated during identically executed experiments of CiOA in male and female C57BL/6J mice. Cartilage damage, osteophyte formation, synovial thickness, and cellularity were assessed by histology on day 56. The association between pain and pathology was investigated, disaggregated by sex.

**Results:**

Differences in pain behavior between sexes were found in the majority of the evaluated pain methods. Females displayed lower weight bearing ability in the affected leg compared to males during the early phase of the disease, however, the pathology at the end stage was comparable between sexes. In the second cohort, males displayed increased mechanical sensitivity in the affected joint compared to females but also showed more cartilage damage at the end stage of the model. Within this cohort, gait analysis showed varied results. Males used the affected paw less often and displayed dynamic weight-bearing compensation in the early phase of the model. These differences were not observed in females. Other evaluated parameters displayed comparable gait behavior between males and females. A detailed analysis of individual mice revealed that seven out of 10 pain measurements highly correlated with OA histopathology in females (Pearson r range: 0.642–0.934), whereas in males this measurement was only two (Pearson r range: 0.645–0.748).

**Conclusion:**

Our data show that sex is a determinant in the link between pain-related behavior with OA features. Therefore, to accurately interpret pain data it is crucial to segregate data analysis by sex to draw the correct mechanistic conclusion.

## Introduction

Osteoarthritis (OA) is a major contributor to chronic pain in adults and is the fourth leading cause of disability worldwide (Murray et al., 2012), affecting 7% of the global population (Hunter, March & Chew, 2020). OA creates an enormous burden on patients and society by severely impacting the workforce (Kontio, Viikari-Juntura & Solovieva, 2020). OA is a progressive disease of the articular joints characterized by cartilage degeneration, ectopic bone formation, and, in approximately 50% of cases, synovitis (Ayral et al., 2005; D’Agostino et al., 2005). However, despite the structural damage, joint pain is the primary OA symptom driving patients to seek medical help.

According to the International Association for the Study of Pain (IASP), pain is described as “an unpleasant sensory and emotional experience associated with, or resembling that associated with, actual or potential tissue damage” (Raja et al., 2020). Pain is a complex phenomenon and there are multiple pain types and modalities according to the nature of the pain stimulus. Pain sensations can vary according to the stimulus and are defined as allodynia (pain provoked by an innocuous stimulus) or hyperalgesia (increased pain perception from a painful stimulus). In OA, joint pain was generally accepted to be nociceptive (often linked to inflammation), in which the nature of the pain is associated with tissue damage in the joint. Presently, nociceptive, inflammatory, and neuropathic pains are all known to occur in OA (Adães et al., 2015; Arendt-Nielsen et al., 2010; Fu, Robbins & McDougall, 2018; Valdrighi et al., 2022). During early OA, patients often describe the exacerbation of pain during movement and relief during rest. As the disease advances, patients describe the pain as either intermitted but generally severe/intense or persistent background pain/aching (Hunter, McDougall & Keefe, 2008; Neogi, 2013). This persistent pain state prevents patients from performing daily activities and taking part in social interactions, altering their physical functioning and mental health, which can lead to personality changes and even depression (Raja et al., 2020).

Pain management is the primary goal for treating OA, since the symptoms may be life-altering, and currently, no approved disease-modifying therapies exist. Despite recent developments, including NGF-targeting strategies, effective pain management is still a challenge. This may be due to a poor understanding of how OA drives pain.

Clinical studies correlating pain and joint changes related to OA (cartilage damage, osteophyte formation, and joint space narrowing) had conflicting results, possibly due to the use of different outcome parameters or large intersubjective variability. OA is also a highly heterogeneous disease from the patient (age, sex, body mass index) and the disease perspective (stage, progression, inflammation) which has prompted the notion of disease phenotypes/subsets (Deveza & Loeser, 2018; Driban et al., 2010). Without the proper distinction of subset/phenotype, it may be very difficult to identify the relevant differences in the disease progression. For example, synovitis is present in 50% of cases and is the only OA feature associated with pain for which there is consensus in literature and which has been clinically associated with OA progression (Ayral et al., 2005) and pain sensitization (Neogi, 2013; Neogi et al., 2016). Sex is another factor that may add to the complexity when trying to determine the cause of pain in OA (Tannenbaum et al., 2019). This factor may have been historically overlooked as a biological variable in preclinical studies. However, when sex was considered, only 42% of the studies analyzed their data by sex (Woitowich, Beery & Woodruff, 2020). However, when aggregated data is analyzed, differences between the sexes may be hidden (Tannenbaum et al., 2019). Preclinical findings from sex-inclusive research have revealed that rodents have remarkably different biological pain pathways between the sexes (Sorge et al., 2011; Sorge et al., 2015). Likewise, in humans, men and women have been shown to respond differently to analgesics (for review, see Packiasabapathy & Sadhasivam, 2018). Women exhibit higher pain sensitivity compared to men in multiple modalities of pain, such as widespread pain and lower pain threshold/tolerance for mechanical pressure, heat, and cold (Bartley et al., 2016).

Even in OA, sex-specific differences are present. OA affects a higher proportion of women than men (almost 2:1), with women also having worse outcomes, partially due to pain (Cho et al., 2010; Glass et al., 2014; Srikanth et al., 2005). However, the majority of preclinical research has been performed on males, with a very limited number of studies investigating sex differences and/or female pain in OA (Contartese et al., 2020). Therefore, our study aims to reveal whether there is a link between pain and OA pathology and if it varies between the sexes.

We compared multiple aspects of pain-related behavior in male and female mice in an OA model with high involvement of inflammation, which is known as collagenase-induced OA (CiOA). We measured mechanical allodynia, pressure-induced hyperalgesia in the affected joint, static weight bearing, and gait analysis (including non-static weight bearing). This is the first preclinical study investigating sex differences in OA pain with synovitis as the main feature (Blom et al., 2004; Ter Huurne et al., 2012).

## Materials & Methods

### Animals

Adult male and female C57BL/6J mice (*n* = 10 per group and sex; Janvier Laboratories, Le Genest-Saint-Isle, France), 10–14 weeks-old, were used in this study. Animals were housed, five per cage, under standard environmental conditions with food and water provided *ad libitum*. Nesting material and two prefabricated refuges (Igloo) per cage were provided as environmental enrichment. Animal order was blindly and randomly assigned by an operator who was not involved in the experiments. During the outcome measurement, blinding was not possible due to obvious phenotypic differences between the sexes. Data analysis was performed blindly. All animal procedures were designed and conducted in accordance with a protocol approved by the Ethical Committee of Animal Research of Radboud University and by the Dutch Central Committee for Animal Experiments (DEC approval n° 2015-0014; AVD10300 2015 115). The experiments followed the guidelines for the Care and Use of Laboratory Animals of the European Union Directive (2010/63/EU) and Dutch regulations (EC2013-235). The experiment was performed in two parts to prevent subjecting the mice to more than two behavioral studies, and a total of 40 mice were used (20 per experiment). As this was an exploratory study, sample size was determined based on previously unpublished data. In addition, 30 mice were used for the CiOA gait experiments database (described below). In total, 70 mice were used in the current study.

### CiOA Model

**Figure 1:**
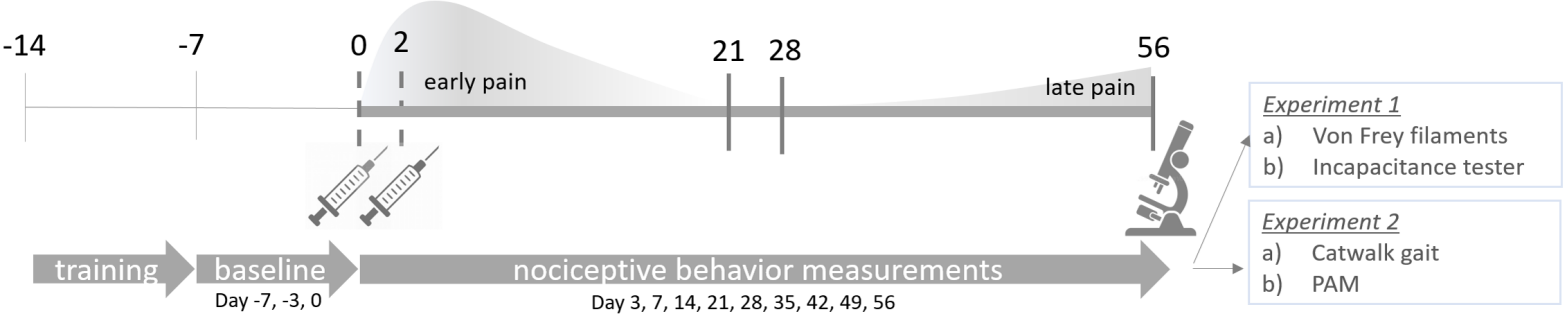
Experimental design. CiOA was induced by two consecutive intra-articular injections of collagenase type VII on day 0 and day 2 in the right knee joint. Training started 2 weeks before model induction (day −14) and baseline behavior was measured at day −7, −3 and 0. Nociceptive behavior was assessed on day 3, 7 and weekly after that, until the endpoint on day 56. Histological parameters were assessed at endpoint. For correlation analysis, early pain is designated as the period between days 0 to 21, while late pain, between 28 to 56. These periods were determined based on the baseline recovery time in which the majority of animals were back to baseline threshold after the initial pain peak for all the evaluated parameters. PAM, Pressure Application Measurement.

Experimental OA was induced as described previously (Van der Kraan et al., 1990). Briefly, mice received two consecutive intra-articular injections in the right knee joint with one unit of collagenase type VII (Sigma-Aldrich, St. Louis, MO, USA) on day 0 and day 2 (Fig. 1). Consecutive injections were performed by the same operator. These injections resulted in local instability of the knee joint, evolving to an OA-like phenotype characterized by cartilage destruction, ectopic bone formation, and chronic synovial activation. Mice were sacrificed by cervical dislocation under isoflurane anesthesia, eight weeks after the first collagenase injection. Control groups were not included, as naïve (Blaney Davidson et al., 2006) or saline-injected (Van der Kraan et al., 1990) animals do not naturally develop changes in joint structure. Throughout the experiment, both the research team and the veterinary staff monitored the welfare of the animals daily. Animal health was monitored by weight, food and water intake, and a general assessment of activity, panting, and fur condition. In all of the experiments the animals did not reach the human endpoint (for detailed humane endpoint criteria, see Supplemental methods).

### Experimental design

Pain-related behavior was evaluated weekly using four different methods to compare multiple aspects of pain between males and females. To prevent a higher degree of agitation from handling, and therefore a higher variability in the outcome, two pain measuring methods per experiment were performed on the same animals. For cohort 1, mechanical allodynia with the von Frey filaments was assessed, followed by weight-bearing in the affected joint with the incapacitance tester (IC), as previously described (Blom et al., 2020). For cohort 2, gait analysis using the Catwalk XT (Noldus, The Netherlands) was performed first as the more passive measurement, followed by mechanical hyperalgesia in the affected joint with the pressure application measurement (PAM; Ugo Basile, Italy) (Fig. 1). Due to the Catwalk apparatus requirements, the nociceptive behavior measurements for the second experiment were performed in a dark room, with red light illumination. For all pain measurements, extreme care was taken to ensure the reliability of the tests. Animals were trained for a period of two weeks before model induction. For the first week, mice underwent a handling/training procedure to get accustomed to the experimental settings. For the second week, three baseline measurements were obtained on alternate days before the start of the experiment: days −7, −3, and day 0. Mice were placed in the procedure room with the person performing the measurements 30 mins prior to the start of the experiment. For standardization purposes, individuals performing the procedure, the starting time, and the procedure order were kept constant throughout the experiment. After model induction, pain-related behavior was measured on days 3, 7, and weekly after that, until day 56.

To compare the pain progression between males and females over time, baseline correction was performed per individual mouse. The mean baseline was calculated and subtracted from each measurement, which set the normalized baseline value at zero. To compare histological parameters and pain per individual mouse, the area under the curve (AUC) was calculated using the raw data and each baseline threshold (min and max values), taking into consideration the peak of nociceptive behavior and whether it was positive or negative. Early and late AUC were calculated in respect of the periods between days 0 to 21, and 28 to 56, respectively, using software Prism 6 (GraphPad software, San Diego, CA, USA). These periods were determined based on the baseline recovery time in which the majority of animals were back to baseline threshold after the initial pain peak for all the parameters evaluated.

### Nociceptive behavior assessment

#### Evaluation of mechanical allodynia (Von Frey test)

To evaluate the mechanical allodynia in the ipsilateral paw, we used a set of eight calibrated von Frey filaments (BioSeb, Vitrolles, France) as previously described (Blom et al., 2020). Animals were individually placed in small Plexiglas cubicles on a metal grid surface and allowed to acclimatize. The filaments were applied perpendicular to the plantar surface of the ipsilateral paw until the fibers bowed and were held for 2 s. The up-down staircase method was used to determine the threshold force necessary for eliciting withdrawal (Dixon, 1991). The grams of force that evoked a positive response more than 50% of the time was determined as described by Chaplan et al. (1994).

#### Evaluation of spontaneous pain (incapacitance and Catwalk tests)

To assess the weight bearing capacity of the hindlimbs, we used an incapacitance tester (Linton Instruments, UK). Mice were placed in a dedicated compartment that positioned the subject with its front paws on a small ramp, thus it was standing on its rear legs. As such, the right and left hind paws were positioned on two separate scales. To ensure reliable results, measurements started when the mouse was in a stable position, as indicated by stable readings for at least 4 s. Five serial measurements were performed per mouse and the percentage of weight on the right limb was calculated, followed by the calculation of the mean per mouse.

To investigate changes in gait parameters by the CiOA induction, and to assess whether differences between males and females were present, gait analysis was performed using the Catwalk-XT (Noldus, Wageningen, The Netherlands). This system consists of a glass plate runway, illuminated with a green light, which the mice are allowed to freely walk across. Underneath the runway, a high-speed camera captures the illuminated footprints and sends them to a computer software. Three valid runs (60% maximal variation in run speed) per mouse were considered a trial. For all data, the trial value was calculated as the mean over the three runs. To measure pain behavior, footprint parameters and spatiotemporal patterns were identified and digitally analyzed (Table 1). Footprint parameters described the use of a single paw, and for that, print area and max contact max intensity from the affected (right hind = RH) and contralateral (left hind = LH) paws were evaluated. The intensity parameter was used to assess the effects of neuropathic pain in the chronic constriction injury model (Vrinten & Hamers, 2003). The spatiotemporal parameters described the dynamic parameters, which included timing and the position of the foot strikes. The simplest units for these patterns were measured as the stand time, the swing time, and the duty cycle. More complex spatiotemporal units involve measures of synchronic support from the contralateral hind paw. The single and initial/terminal dual stances are used for gait analysis in pain models (Blom et al., 2020; Coulthard et al., 2002; Coulthard, Simjee & Pleuvry, 2003). These parameters quantify gait modifications that can be linked to limping in humans.

**Table 1:** Overview of Catwalk XT gait parameters. Description and direction of parameter translating into pain-related behavior.

Parameter	Description	Pain-related behavior^*^
(Foot) print area	mean width x length for a paw	↓
Max contact max intensity	measure of the weight support put on a paw on the glass plate	↓
Stand time	duration of paw contact with the ground	↓
Swing time	time in which the paw is in the air until next stand	↑
Duty cycle	the ratio of the stand time as a percentage of the step cycle (stand + swing)	↓
Single stance	stand time in which the contralateral hind does not touch the plate, with the hind support laying only in the affected limb	↓
Initial dual stance (IDS)	measures the overlap period at the beginning of the RH step cycle in which there is still a support from LH	↓
Terminal dual stance (TDS)	duration in the end of the support of RH that overlap with the beginning of contact for LH	↑

**Notes.**

* related to the affected paw (RH): ↓ (lower) or ↑ (higher) number corresponds to more pain.

#### Evaluation of mechanical hyperalgesia (PAM test)

Hypersensitivity in the affected joint was assessed using the pressure application measurement (PAM) device (Ugo Basile) as previously described (Barton et al., 2007). Briefly, mice were gently restrained by hand and the small force transducer (dedicated to mouse measurements) was placed on the medial side of the knee joint. Squeeze force was gradually applied, guided by the PAM software, at a constant rate (30 gf/second). The response was recorded when the mouse tried to withdraw or responded with agitation. When no response occurred, a maximal of 450 gf was applied. Two measurements, at least 5 min apart, were recorded per animal and the mean withdrawal force per mouse per day was calculated.

#### Histological analyses and scoring of knee OA pathology

Mice were euthanized by cervical dislocation under isoflurane anesthesia on day 56 after induction of CiOA. The knee joints of the test animals were isolated and fixed in 4% formaldehyde, decalcified in formic acid, and subsequently dehydrated and embedded in paraffin. Coronal sections were cut at 7 µm and mounted on coated slides. One set of slides was stained with Safranin O/Fast Green (SafO) and another with hematoxylin and eosin (HE). Osteophyte formation, cartilage damage, joint dislocation, and synovial changes were assessed in the below-mentioned sections.

#### Osteophyte formation

We assessed the locations of ectopic bone formation as previously described to investigate the formation of osteophytes (Blaney Davidson et al., 2007). Briefly, every joint stained with HE was scored for osteophyte formation at all locations on both lateral and medial sides, as well as the central area.

#### Cartilage damage (OA score) and joint dislocation

Cartilage destruction was determined in five sections per knee joint stained with Safranin O/Fast Green using a histologic scoring of murine OA (Glasson et al., 2010). Briefly, the OA score was the assessment of the depth progression of OA into the cartilage. The OA score from the five sections was determined per location (medial and lateral sections in both the tibia and femur) and the mean of each location per animal was calculated, with a maximum grade per location of 30 (5 × 6). The total OA score was determined by the average of the four locations together (with a max value of 30). During this scoring, the presence of dislocations was also noted per animal. Animals with patella dislocation were included in the analysis as it may contribute to pain-related behavior.

#### Synovial thickness and cellularity

For changes in the synovium, the thickness and cellularity were assessed in knee joint sections stained with HE. Images were acquired with the Pannoramic P1000 (3DHistech, Budapest, Hungary) and analyses were performed using Fiji/ImageJ (Schindelin et al., 2012). Synovial thickness was measured as the width from the bone margin to the capsule in the parapatellar recesses at the medial and lateral sides at three positions per section. Measurements were performed in three sections per knee. In total, 18 measurements of synovial thickness per knee were obtained, which were averaged to reach a single value per knee joint. The same three sections per knee were used for synovial cellularity assessment. The image was prepared using Fiji and following these steps: color deconvolution, HE, blue color was selected because the cell nucleus was visible and Li threshold and watershed were applied. The synovial area was selected as the region of interest and the area covered by the cell nucleus was calculated as a percentage of the total area. A total of six measurements per knee were acquired and averaged, resulting in one single value per knee joint.

#### Database on CiOA gait experiments for retrospective analysis

For validation purposes, unpublished experiments from our database on CiOA gait analysis with the Catwalk XT were re-analyzed according to the strategy used in the current study. Experiments were conducted according to Dutch law and approved by the Dutch Central Animal Experimentation Committee (projects 2011-227 and 2014-165). Deviations from the current experiment (Table 2) included a different endpoint(day 42), and the evaluation of only gait analysis and cartilage damage. One experiment included male and female mice (Dataset 1), while the other had only females (Dataset 2). Moreover, one intra-articular collagenase injection was used for model induction, and one baseline measurement was performed for the gait analysis in an experiment using both male and female mice. Ten mice per group were used for both experiments.

**Table 2:** Overview of experiments: protocol similarities and differences.

Experiment	Cohort 1	Cohort 2	Dataset 1	Dataset 2
Sex	M/F	M/F	M/F	Only F
animals per group (n)	10	10	10	10
collagenase injections (n)	2	2	1	2
Endpoint (day)	56	56	42	42
Pain measurements	VF + IC	Catwalk + PAM	Catwalk	Catwalk
Baseline measurement (n)	3	3	1	3
Histology outcome	Cartilage damage + Osteophyte number	Cartilage damage + Osteophyte number	Cartilage damage	Cartilage damage

**Notes.**

M males F females VF Von Frey test IC Incapacitance testers PAM Pressure Application Measurement

#### Heatmap and the association between histological characteristics and nociceptive profile

A heatmap was developed based on Pearson coefficient values (r) to provide an overview of the associations between the histological parameters of OA and the different pain parameters (AUC). The following histological parameters were placed horizontally: cartilage damage, dislocation, osteophyte number, synovial thickness, and synovial cellularity. The pain variables (AUC) were distributed vertically. The coefficient values were displayed for significant correlations, followed by the level of significance. For non-significant correlations, the r values were omitted. All coefficient values were color-coded as bluefor positive correlations, yellow for r close to zero, and red for negative correlations. Pearson coefficient values range from +1 to −1, with +1 as a perfect positive correlation, 0 as no correlation, and −1 as a perfect negative correlation.

### Statistical analyses

Results are presented as mean ± standard error of the mean (SEM). The normality of all data was assessed using the Shapiro–Wilk test. Nociceptive behavior was analyzed by repeated measures (RM) two-way ANOVA, followed by the Bonferroni multiple comparison post-test for comparison between sexes and to the baseline. The *P* value was corrected for multiple comparisons. Male *versus* female differences in histology were assessed using the Mann–Whitney test. All statistical analyses were performed using the Prism 6 software (GraphPad software, San Diego, CA, USA), except for the correlation tests which were analyzed by Pearson correlation analysis using SPSS (IBM Corp., Armonk, N.Y., USA). Significant differences were considered as *p* < 0.05.

## Results

### Mechanical allodynia and static weight-bearing symmetry following CiOA (cohort 1)

**Figure 2:**
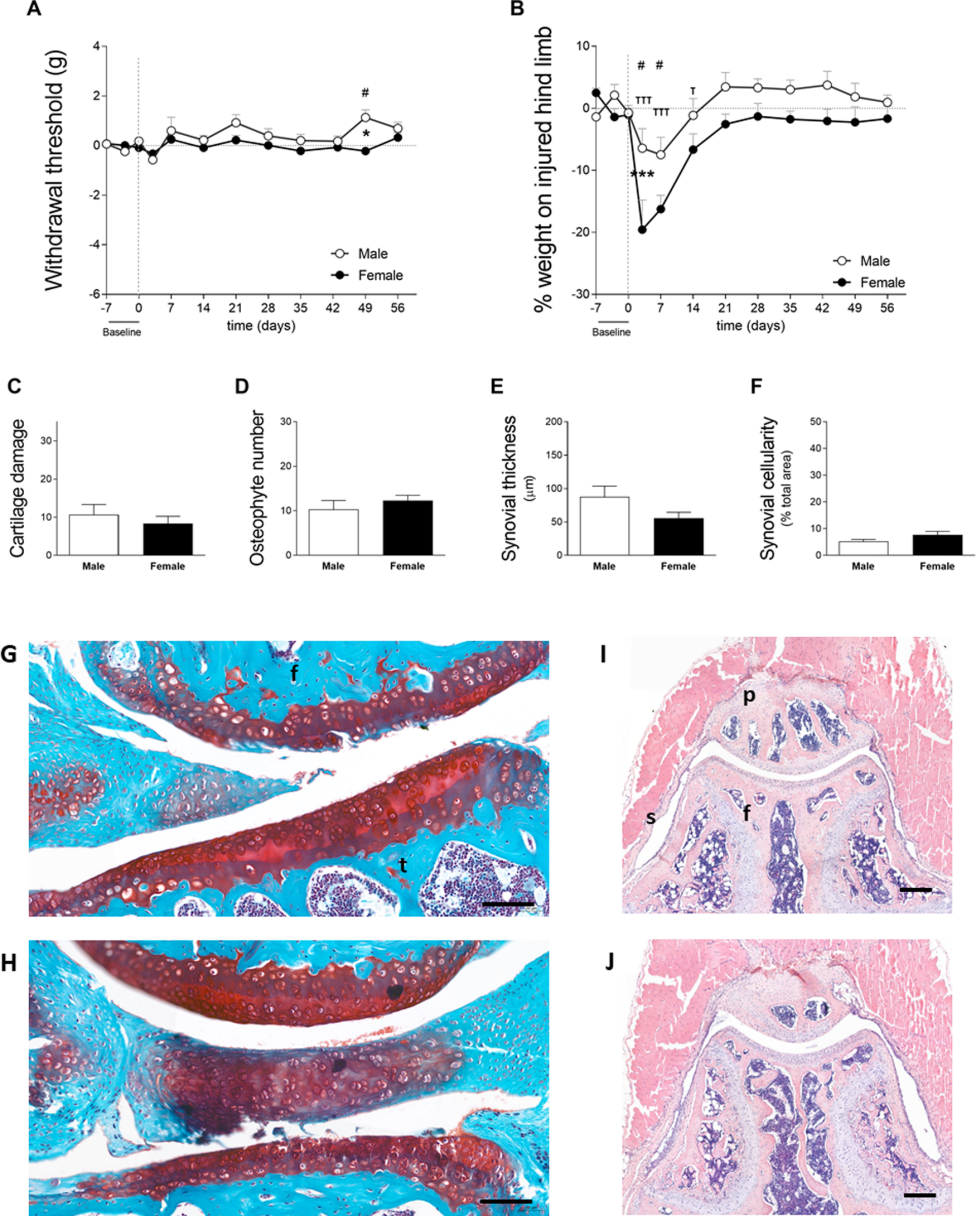
Females exhibit a more dramatic decrease in weight bearing during the early phase of OA (cohort 1). (A) 50% paw withdrawal threshold measured with the von Frey filaments (in grams). Values are corrected for individual baselines. Negative values indicate mechanical allodynia in the affected joint. (B) Weight bearing on the right hind limb measured with the incapacitance tester. Values are corrected for individual baselines. Negative values indicate lower weight bearing in the affected joint. (C) Histologic scoring of total cartilage damage. (D) Total number of locations with ectopic bone formation. Synovial (E) thickness and (F) cellularity. All histological parameters were measured at end of the experiment, on day 56. Representative images of cartilage damage in the femoral condyle and tibial plateau with safraninO/Fast Green staining in (F) and (G) (scale bar = 100 µm) and synovial changes in the patellofemoral space with HE staining scale in (I) and (J) (scale bar = 200 µm) of males and females, respectively, knee joint cavity sections; f, femur; t, tibia; p, patella and s, synovial membrane. For pain measurement, RM two-way ANOVA and Bonferroni’s multiple comparisons test. * (black asterisk) male *vs* female comparison; *τ* female to baseline and # male to baseline comparison. For the remaining graph, the Mann–Whitney test. * *p* < 0.05 and *** *p* < 0.001. *N* = 10 mice per group. Data are expressed as mean ± SEM.

In cohort 1, mechanical allodynia was assessed with the ipsilateral foot pad using the von Frey filaments; however, this sensation was not observed in male or female subjects (Fig. 2A). Significant results were observed in males on day 49 compared to baseline, as well as in males compared to females; however, these differences were not indicative of mechanical allodynia. Within the same cohort, weight bearing asymmetry was more obvious during the early phase of CiOA in both males and females when compared to the later phase. Two-way ANOVA analysis showed that sex had a significant effect (*p* = 0.0118) with females displaying lower weight bearing in the affected hind limb than males.

Both sexes avoided loading support in the ipsilateral leg on days 3 (M 6.4 ± 9.9; F 19.6 ± 15.1) and 7 (M 7.5 ± 8.9; F 16.3 ± 7.0), with only females still showing impairment on day 14 (M 1.1 ± 8.6; F 6.7 ± 8.0). When comparing the magnitude of response between males and females, females displayed a lower weight bearing capacity on day 3. Females avoided bearing 20% of their weight on the affected limb compared to 6% in males (*p* < 0.001, Fig. 2B). Despite the differences in weight bearing, histological analysis of the joint at endpoint showed no significant differences between males and females in cartilage damage (M 10.7 ± 8.0; F 8.3 ± 5.8, Fig. 2C), osteophyte formation (M 10.3 ± 6.0; F 12.3 ± 3.6, Fig. 2D), and synovial alterations (M 87.6 ± 51.4; F 56.0 ± 27.3, Fig. 2E and M 5.2 ± 2.2; F 7.7 ± 3.8, Fig. 2F). Three males had joint dislocations compared to one female. These results indicate that females may experience more pain during the early phases of the disease or have different compensatory behaviors to relieve the affected joints, despite a similar degree of pathology at the endpoint.

### Mechanical hypersensitivity in CiOA mice (cohort 2)

**Figure 3:**
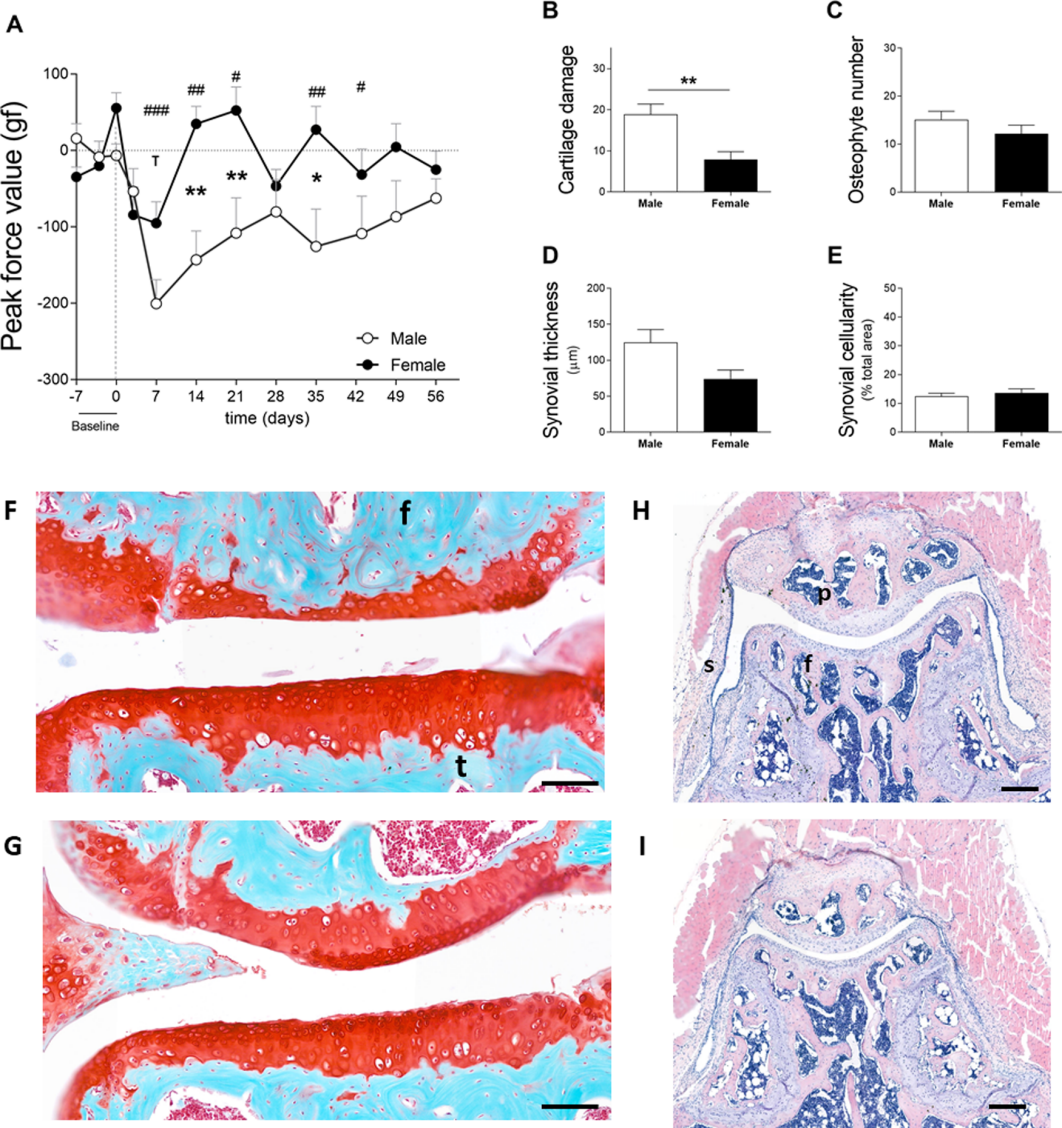
Males experience increased hyperalgesia in the affected joint compared to females but also showed more pronounced OA (cohort 2). (A) Hypersalgesia in the affected joint measured with the peak force value using the PAM. Values are corrected for individual baselines. Negative values indicate lower pressure tolerance in the affected joint compared to baseline. (B) Histologic scoring of total cartilage damage. (C) Total number of locations with ectopic bone formation. Synovial (D) thickness and (E) cellularity. All histological parameters were measured at the end, on day 56. Representative images of cartilage damage in the femoral condyle and tibial plateau with safraninO/Fast Green staining in (F) and (G) (scale bar = 100 µm) and synovial changes in the patellofemoral space with HE staining scale in (H) and (I) (scale bar = 200 µm) of males and females, respectively, knee joint cavity sections; f, femur; t, tibia; p, patella and s, synovial membrane. For pain measurement, RM two-way ANOVA and Bonferroni’s multiple comparisons test. * (black asterisk) male *vs* female comparison; *τ* female to baseline and # male to baseline comparison. For the remaining graph, the Mann–Whitney test. * *p* < 0.05 and ** *p* < 0.01. *N* = 10 mice per group. Data are expressed as mean ± SEM.

In cohort 2, male and female mice exhibited increased sensitivity in the affected joint on day 7 after the induction of OA. Males and females reacted to a force of 200.6 ± 99.3 gf and 95.4 ± 89.0 gf less than before model induction, respectively (Fig. 3A). Females recovered to near baseline force values by day 14. However, in males, the affected joints remained in a hypersensitive state during the full course of CiOA. Two-way ANOVA analysis showed that sex (*p* = 0.0063), time (*p* = 0.0008), and the interaction between them (*p* = 0.0037) had a significant effect on pain levels. The differences between males and females were present on days 14 (*p* < 0.01), 21 (*p* < 0.05), and 35 (*p* < 0.01), suggesting that males experienced increased sensitivity in the affected joint when compared to females. As opposed to the previous experiment, histological analysis revealed that males presented more cartilage damage as measured by the OA score (Fig. 3B, M 18.8 ± 8.0; F 7.8 ± 6.1; *p* = 0.0051), as well as more joint dislocations (six males *versus* two females) on day 56. No differences were observed in the number of osteophytes (M 15.1 ± 5.6; F 12.2 ± 6.0; Fig. 3C). Males tended to have a thicker synovium (124.5 ± 57.4 *versus* 74.0 ± 39.3, Fig. 3D) than females; however, this difference was not significant ( *p* = 0.063). The synovial cellularity remained similar between the sexes (M 12.5 ± 3.4; F 13.7 ± 4.4; Fig. 3E). These findings demonstrate that the higher and more prolonged hypersensitivity of the affected joints in males coincided with more severe histological parameters.

### Spontaneous pain during voluntary gait following CiOA (cohort 2)

**Figure 4:**
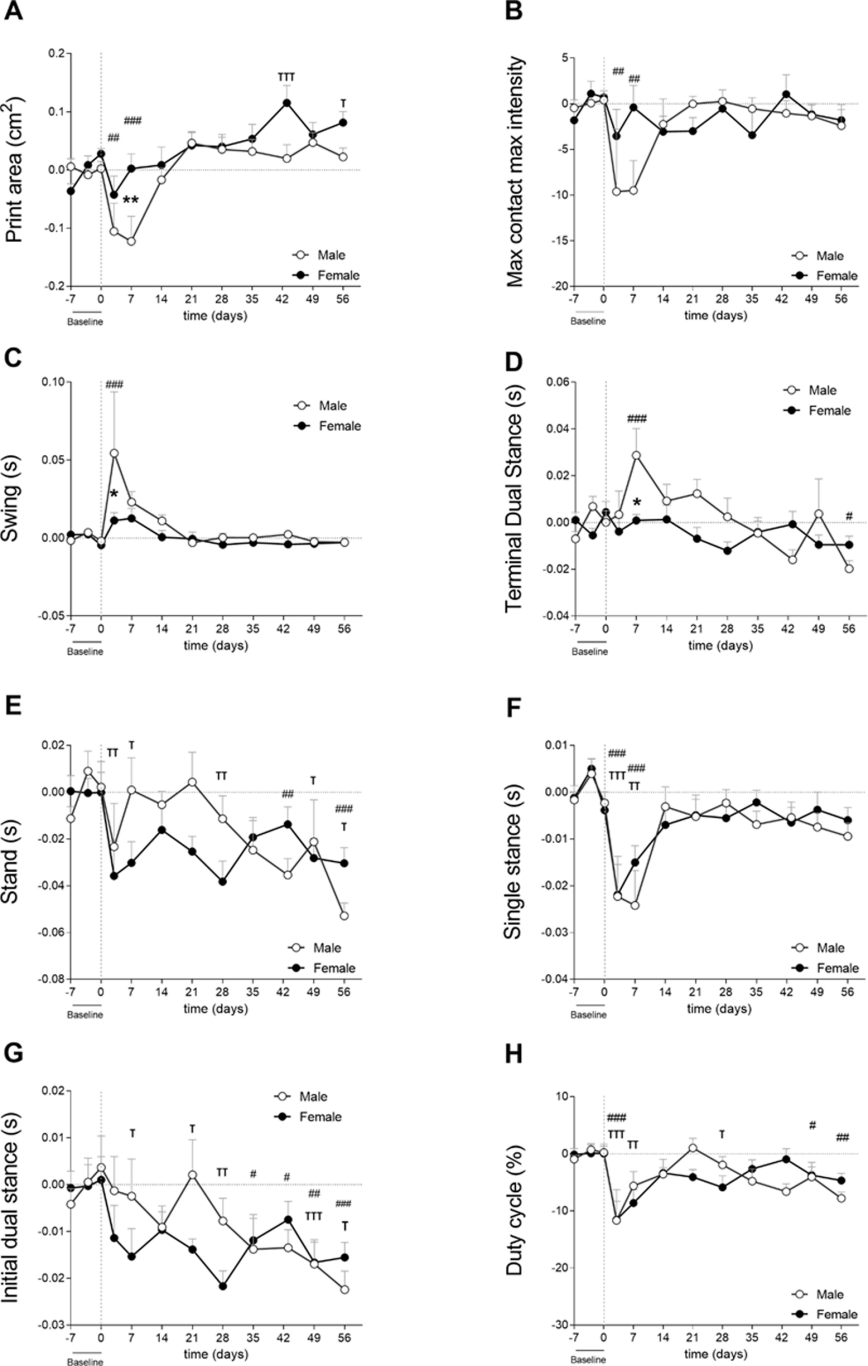
Gait analysis showed parameter-dependent differences between sexes (cohort 2). Gait was evaluated using the Catwalk system. For all parameters, values are corrected for individual baseline. (A) RH Print area. (B) RH Max contact max intensity. (C) RH Swing. (D) RH terminal dual stance. (E) RH Stand. (F) RH Single stance. (G) RH Initial dual stance. (H) RHDuty cycle. RM Two-way ANOVA and Bonferroni’s multiple comparisons test. * (black asterisk) male *vs* female comparison; *τ* female to baseline and # male to baseline comparison. * *p* < 0.05, ** *p* < 0.01 and *** *p* < 0.001. *N* = 10 mice per group. Data are expressed as mean ± SEMD. RH, Right hind.

Cartilage damage was shown to be more severe in males, as mentioned in the previous section (Fig. 3B—Cohort 2). Males used the affected paw less often during early CiOA compared to baseline (Fig. 4A, D3, *p* < 0.01; D7, *p* < 0.001), which translated into a smaller print area on days 3 and 7. Males displayed a smaller print area than females on day 7. In addition, males also showed decreased weight support compared to baseline (max contact max intensity; Fig. 4B, D3, and D7, *p* < 0.01), suggesting mechanical allodynia. This impairment recovered to baseline values by day 14. In contrast to the males, females did not show significant changes compared to baseline in these footprint parameters at the early time points. However, females did show compensatory behaviors in the contralateral paw print area (Fig. S1A) over multiple time points during early and late phases. Two-way ANOVA analysis showed that sex had a significant impact on the affected paw print area values, with males displaying a lower print area than females (*p* = 0.0424). For the max contact max intensity, no significant effect of sex was observed.

Spatiotemporal parameters indicated that males experienced more nociceptive behavior in the early phases as indicated by a significantly longer swing time of the affected paw compared to females on day 3 (Fig. 4C). Males also displayed earlier weight bearing compensation by the contralateral hind paw (TDS; Fig. 4D) on day 7 (*p* < 0.05). Stand (Fig. 4E), single stance (Fig. 4F), and IDS of the affected hind limb (Fig. 4G) did not show significant differences between males and females. However, stand, IDS, and TDS two-way ANOVA analysis demonstrated that sex alone did not have an influence, but when interacting with time, the results were significant (*p* = 0.0065, *p* = 0.0191, and *p* = 0.0035, respectively), indicating that pain development over time in CiOA is different between males and females. The duty cycle, which is the ratio of the stand phase of the whole step cycle was significantly smaller in the CiOA limb in both males and females compared to baseline on day 3 (Fig. 4H, *p* < 0.001). Males had a significantly decreased duty cycle on days 49 (*p* < 0.05) and 56 (*p* < 0.01); however, no significant sex differences were observed.

Most of the changes in CiOA were observed in the early stages of the disease; however, CiOA seems to have promoted comparable adaptations in gait strategies on day 56 in males and females. Both sexes displayed a lower stand time on the affected paw (Fig. 4E), as well as a decreased IDS.

Gait analysis from the present study indicated that differences between males and females may be parameter-dependent, indicating differences in nociceptive-related compensatory mechanisms.

### Association between nociceptive behaviors and histological parameters

**Figure 5:**
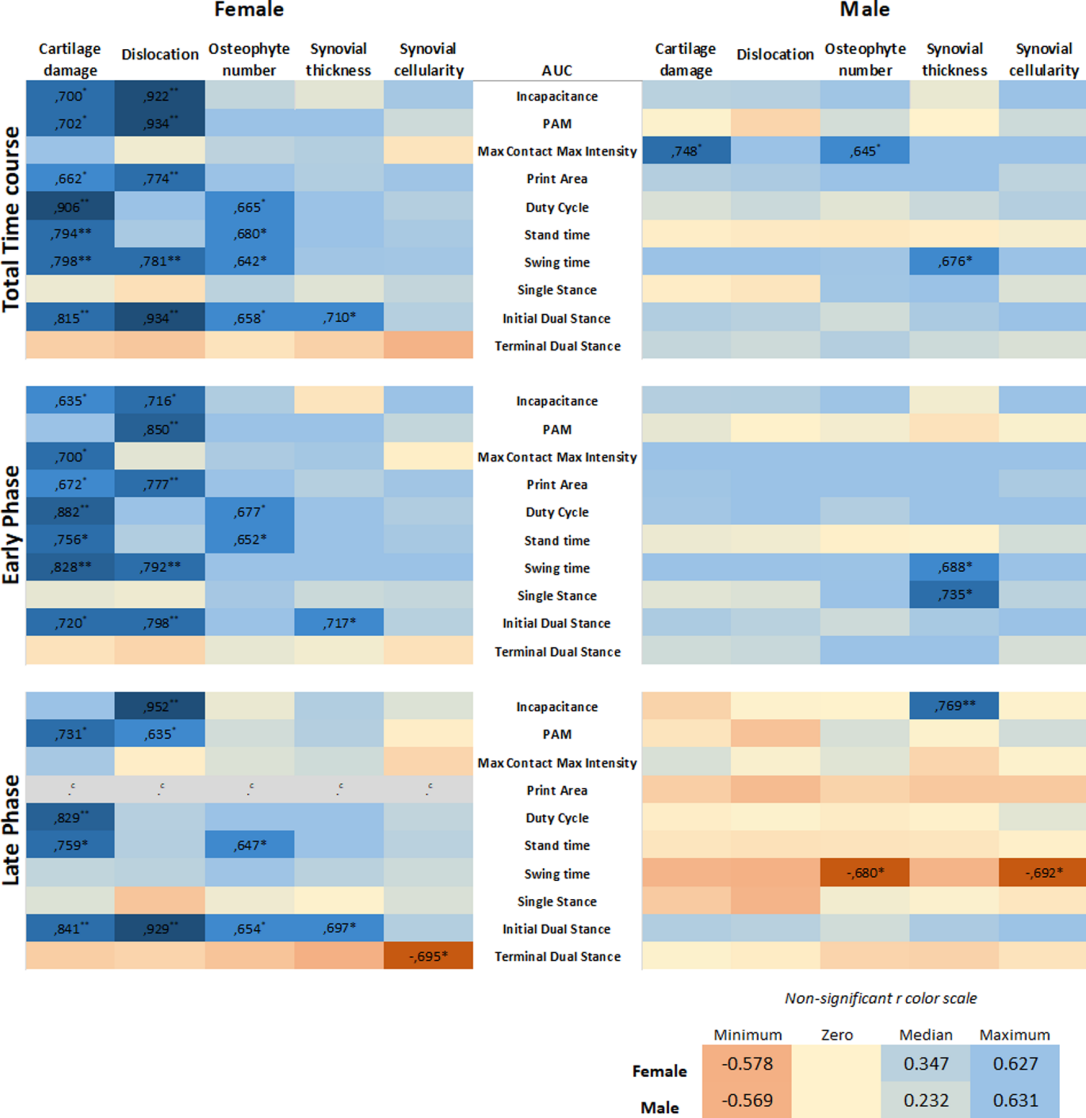
Level of pain highly correlates to histological parameters only in females. Heatmap based on the Pearson coefficient values (r) between OA histological parameters and the different AUC pain parameters during the whole time course (top), early phase (middle), and late phase (bottom). Coefficient values are displayed for significant correlations followed by the level of significance (*). For non-significant correlations, the r values were omitted. All coefficient values were color coded as blue for positive correlations, yellow for r close to zero, and orange for negative correlations. Pearson coefficient values range from +1 and −1, with +1 as a perfect positive correlation, 0 as no correlation, and −1 as a perfect negative correlation. * *p* < 0.05 and ** *p* < 0.01. For specific *P* values, see Table S1. *N* = 10 mice per group. ^*c*^. Cannot be computed because at least one of the variables is constant.

To investigate whether sex is a determinant in the link between histological parameters and pain, data were analysed on an individual mouse level, disaggregated by sex. The area under the curve (AUC) of each pain parameter per individual mouse was calculated and used in correlation analyses to examine the relationship between the OA parameter and pain behavior. The number of significant correlations was compared between males and females. The AUC was first calculated for the entire time course of CiOA, since disease pathology at the endpoint is a result of events that occurred throughout the duration of the disease. This correlation analysis yielded 17 positive correlations for females, with r values between 0.934 and 0.642 (Fig. 5, Pearson r with significant levels *p* < 0.05 and *p* < 0.01), distributed over seven out of the 10 pain parameters. Males displayed only three positive correlations (r values: 0.645, 0.676, and 0.748, with significant levels *p* < 0.05), within two pain parameters, max contact max intensity, and swing time.

Most of the pain-related behavior was observed in the early phase; therefore, the correlation analysis was divided by the AUC of the early (day 0–21) and late phases (day 28–56). Remarkably, in the early phase, females showed 15 positive associations, with r values between 0.635 and 0.882, distributed over eight of the 10 pain parameters. The pain was associated more closely with hard tissue changes (cartilage damage and dislocation, and osteophyte number), in contrast to one positive association with synovial thickness. Males showed no associations between pain and hard tissue changes and displayed only two positive correlations with the synovial thickness. In the late phase comparison, females also showed positive associations; however, these were fewer than in the early phase, with 10 significant correlations in five of the 10 pain parameters. Females also displayed one negative correlation. Males showed only two negative correlations, suggesting that the number of osteophytes and synovial cellularity in the late phase are inversely proportional to swing time alterations.

Positive correlations indicate that the increase in two variables is proportionally related; and, these results indicate that the level of pain-related behavior highly correlates to histological parameters in females, while in males, this correlation was nearly absent. The complete data of the calculated correlations and corresponding *p*-values per parameter are shown in the Table S1.

### Retrospective correlation analysis of pre-existing CiOA data

**Figure 6:**
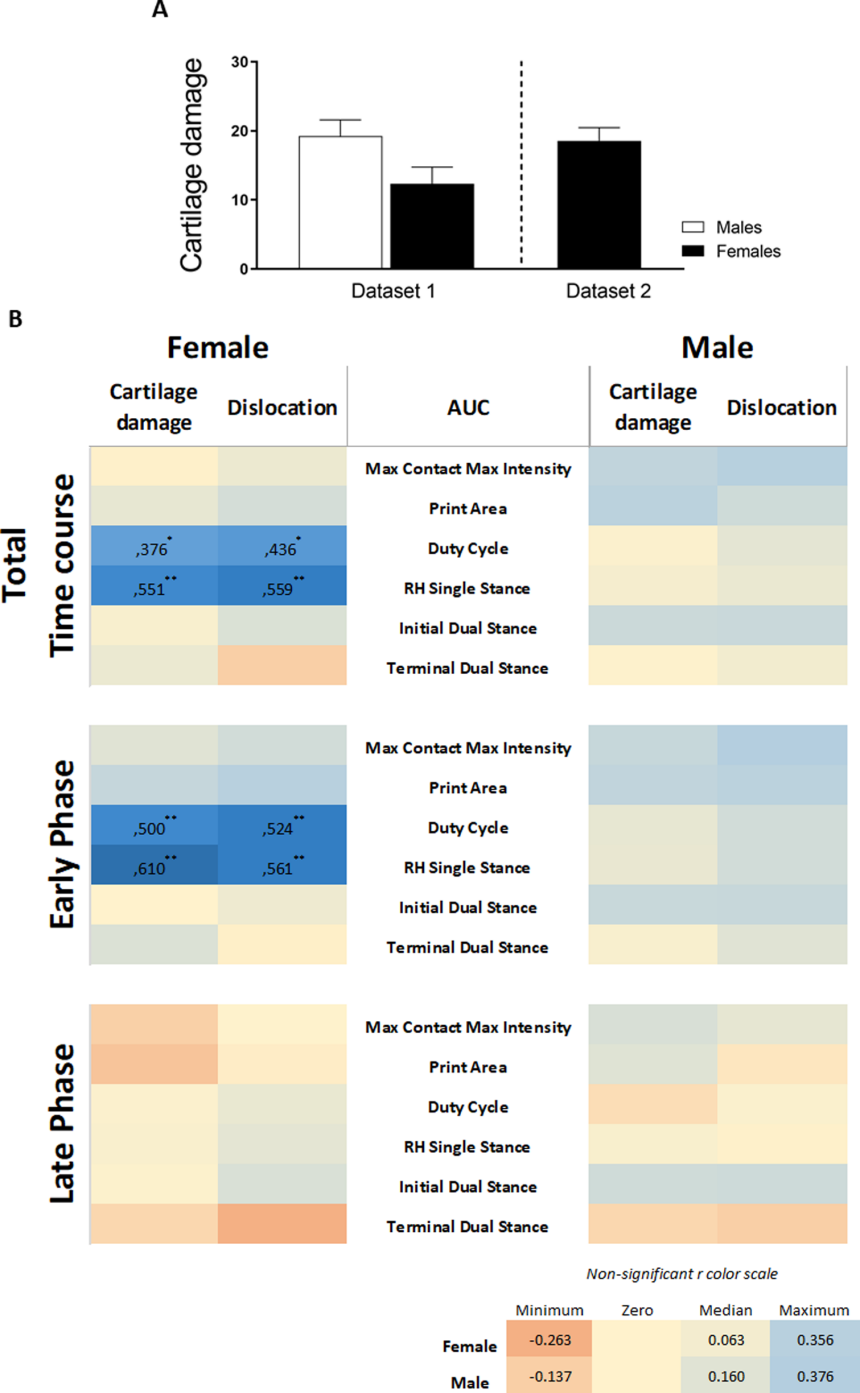
Retrospective data analysis confirmed the association of pain behavior and histological parameter in females. (A) Histologic scoring of total cartilage damage and (B) heatmap based on the Pearson coefficient values (r) between OA histological parameters and the different AUC pain parameters during the whole time course (top), early phase (middle), and late phase (bottom). Coefficient values are displayed for significant correlations followed by the level of significance (*). For non-significant correlations, the r values were omitted. All coefficient values were color coded as blue for positive correlations, yellow for r close to zero, and orange for negative correlations. Pearson coefficient values range from +1 and −1, with +1 as a perfect positive correlation, 0 as no correlation, and −1 as a perfect negative correlation. * *p* < 0.05 and ** *p* < 0.01. For specific *P* values, see Table S2. Histological data are expressed as mean ± SEM. *N* = 20 male mice and 30 female mice.

We re-analyzed pre-existing data (Table 2) on CiOA gait analysis using the catwalk according to the above-described strategy to validate our findings. Cartilage damage assessed by histopathological scoring displayed a high degree of variation between sexes and experiments. For the experiment including males and females (Dataset 1), males showed slightly more severe cartilage damage compared to females, however, the difference was not significant (*p* = 0.0524, Fig. 6A). Males also presented more knee joint dislocations than females (six in males and three in females). In the experiment with only females (Dataset 2), cartilage damage and the number of animals with dislocation (seven out of 10 animals) were similar to the males in experiment 2 (Fig. 3). When combining the data from the pre-existing dataset and the experiments (females: *n* = 30 and males: *n* = 20), the correlation analysis from the AUC of the pain parameters and cartilage damage and dislocations resulted in significant positive correlations in females only. Females displayed four moderately positive correlations with r values between 0.376 and 0.559 (Fig. 6B, Pearson r with significant levels *p* < 0.05 and *p* < 0.01) for females, distributed over two of the six evaluated pain parameters. In evaluating the effects of the early and late phases of the disease, females showed four positive correlations, with higher r values, between 0.500 and 0.610 in the early phase (day 0–21) (Fig. 6B, Pearson r with significant level *p* < 0.01). The late phase correlations showed no associations between pain and damage in either males or females, with males showing no significant correlations in any of the periods or parameters investigated.

The inclusion of data from two previous studies in CiOA, which were subjected to the same association analysis, substantiated our primary findings that the association between the histological parameters of OA is present in females and is nearly absent in males. The complete data of the calculated correlations and corresponding *p*-values per parameter are shown in Table S2.

## Discussion

In this study, we investigated the link between pain and histological parameters and whether there is difference between the sexes. Multiple aspects of pain were studied in male and female mice during the course of CiOA. For both sexes, CiOA mainly triggered pain-related behavior during the early phase (until day 21). Gait asymmetry measured with the stand and initial dual stance for both sexes and terminal dual stance and duty cycle in males only, was observed during the late phase. Changes in these parameters can be comparable to limping in humans. Differences in pain behavior between males and females were found in most of the evaluated pain aspects, with the exception of mechanical allodynia measured with the von Frey, which was not observed in this experiment. Females displayed lower weight bearing capacity in the affected leg compared to males (cohort 1—Fig. 2) with a similar degree of histological parameters. In an experiment with more cartilage damage and joint dislocation in males, these mice displayed increased hyperalgesia in the affected joint as compared to females (cohort 2—Fig. 3). Interestingly, gait analysis from the present study suggests that differences between males and females are parameter-dependent (Fig. 4) with different pain-related compensatory mechanisms. However, because of the extent of the differences in pathology between the sexes, it cannot be concluded that this is due to a sex-related effect.

The most striking results came by correlating pain with OA histopathology on an individual mouse level. This showed that in females OA damage on hard tissues with changes including cartilage damage and osteophyte formation was strongly correlated with joint pain-related behavior; whereas in males this relationship was nearly absent. The correlation between cartilage damage and dislocation with pain in females was confirmed by an analysis of pre-existing datasets of CiOA gait analysis. This also allowed direct comparison between males and females having a similar degree of cartilage damage and number of joint dislocations, which still showed that the correlation between cartilage damage and pain behavior was stronger in females and weaker in males. Thus, the observed differences between males and females regarding this correlation were not due to a difference in pathology between the sexes and strongly suggest a difference in the pain pathways between sexes (Fig. S2). This was further substantiated by the fact that pain in males seems to be associated with increased synovial thickness at day 56, and the absence of hyperplasia suggests that either joint inflammation was in remission and/or a fibrotic process was ongoing. It is well known that synovial fibrosis contributes to pain and joint stiffness in OA (for review, see Remst, Blaney Davidson & Van der Kraan, 2015).

The observed differences in the association between pathology and pain behavior between sexes may be linked to the adaptive cost of the pain behavior itself. From the evolutionary perspective, males and females developed adaptive functions aiming at reproduction, resulting in unique behavior between them. Among these, males evolved towards the expression of dominance or power dynamics and resource acquisition, leading to a different relation to stress and pain than females (Seifarth, McGowan & Milne, 2012). These distinct behavioral patterns between sexes are, in part, a consequence of different sex hormones (for review see Adkins-Regan, 2012; Goldey & Van Anders, 2015; Wallen & Zehr, 2004). This may lead to different pain pathways and might be intertwined with the differential activation of the stress hormone systems between males and females (Butler & Finn, 2009). In this context, the distinct roles of sex hormones are shown, with androgen being mainly an anti-inflammatory hormone (Cai et al., 2016) and are estrogen protecting cartilage (Bay-Jensen et al., 2013), as is being revealed in current epidemiological studies (Hughbanks et al., 2021; Jones & Jørgensen, 2020). These mechanisms could play an additional role in the effect that sex has on the link between OA damage and pain, warranting further investigation.

Despite our increasing knowledge about sex differences, until recent years, sex was not recognized as a biological variable and the preference for males in preclinical research was a generalized standard (Woitowich, Beery & Woodruff, 2020), including in OA research (Contartese et al., 2020). Many studies did not mention the sex of their subjects, being cells or animals. If both sexes were included in a study, data analysis and reports were rarely performed by sex, which ignores the modifier effect that sex may have. This is the first study that comprehensively evaluated and compared CiOA pain in males and females. However, past study from our group had shown that sex difference in the prevalence of CiOA was present (twice as high in males as in females) (Van Osch et al., 1993). In the current study, differences were sometimes observed in histological severity, not in prevalence. Moreover, the CiOA induction protocol has since been modified by intraarticular collagenase injection on two alternate days rather than once (Blom et al., 2004). Previously pain in CiOA had been studied using the incapacitance tester in males (Lee et al., 2020) or with both sexes without reporting data per sex (Cook et al., 2012; Lee et al., 2018). In earlier studies, weight distribution was significantly altered only after day 20 and did not show the early phase pain, as observed in the present study. A possible explanation for this discrepancy may be the difference in the age of the animals at the time of OA induction. Previous studies used 7 week-old mice whereas we used 10–14 weeks old mice, which by human comparison standards, would be between adolescents (puberty) and mature adults (adulthood), respectively. This means that their mice were still in an active growth phase and changing sex-hormone levels in contrast to our situation of full-grown joints and stable hormone levels. The sex differences in pain were recently investigated in traumatic OA models. In studies using a destabilization of the medial meniscus (DMM) model, joint damage was more extensive in male mice compared to females. However, there was a subtle sex difference in the exhibited pain behavior (Hwang et al., 2021; Von Loga et al., 2020). A study using the meniscal transection-induced OA (MMT) model (Temp et al., 2020) in 8–10 week-old mice showed that males developed more severe cartilage damage than females; and sex differences were pain-modality and time-dependent, corroborating the present study. However, in the MMT model, the correlation between knee damage and the pain was absent in both males and females. This discrepancy in correlation with the results of the present study may be due to the degree of inflammation involved in the models: there was no-to-low involvement in the MTT and high involvement in the CiOA. The age of mice may also have been a determinant, as the inflammatory response of adolescent mice is weaker than that of adult mice. These results corroborate the findings of the previously mentioned clinical studies, in which synovitis is the only OA parameter that correlates with OA progression (Conaghan et al., 2010; Wang et al., 2018) and pain sensitization (Neogi, 2013; Neogi et al., 2016).

The positive association between pain sensitization and synovitis is usually described as moderate (Guermazi et al., 2014). In the present study, pain and CiOA damage showed a high degree of correlation in females only, and was nearly absent in males. When the current data was analysed with males and females combined, then the pain-OA damage correlation was also moderate (Fig. S3). These findings suggest that disaggregating the analysis per sex is necessary for studies connecting pain-pathology. Recent clinical cohort data on OA pain and obesity performed a sex-stratified analysis and the outcome corroborates our hypothesis. Multiple clinical and biological factors were associated with pain (OAKHQOL, WOMAC and VAS scores) in females, including radiographic severity (Kalgreen-Lawrence stage). These associations were nearly absent in males, with their severity only being associated with the VAS score (Sellam et al., 2021).

The findings of this study have some limitations. First and foremost are the structural differences between the sexes. To account for the possible differences in hip biomechanics in weight bearing between the sexes, raw data was transformed into percentage of change (as compared to baseline) to establish comparisons between females and males. Another possible limitation to consider is the fact that growth plates in rodents remain open, despite achieving sexual maturity, which may explain, in part, some discrepancies between the observations made in the early and late phases of CiOA (Aigner et al., 2010). In addition, pathology in CiOA can vary between experiments and this cannot be controlled. The differences in the severity of CiOA pathology in the present study prohibit ideal comparison between sexes, as pathology is a confounding factor. Nonetheless, to ensure proper conclusions, the present study only made claims about sex differences in pain between cohorts (in one experiment, female mice showed more pain behavior, while in the other experiment, males exhibited more pain), but also highlighted the differences in the severity of OA pathology. Despite the differences in histopathology, the association between nociceptive behavior in the early phase and histological parameters at endpoint were strongly linked in females. Furthermore, it was anticipated that a chronic pain phase would be reached if the experiment had a longer duration than is standard within our laboratory (day 42). Therefore, extrapolating conclusions to those similar in patients with OA is not possible. In addition, the use of gait and weight bearing experiments to determine pain levels must also consider the interference of functional impairment due to altered biomechanics (Xu et al., 2019).

Nevertheless, despite the severe damage to the joints at the endpoint on day 56, the absence of chronic pain over multiple pain modalities may also provide an opportunity to investigate factors that are present or absent in the progression of CiOA which prevents pain from becoming chronic. Clinically, the findings from the present study provide hints of sex-based influences in the relationship between early joint pain and late OA damage. This generates a testable hypothesis for larger sample sizes, as well as the need to identify the subsets for which OA subsets/phenotypes are applicable. Based on the results of this study and once early pain was more prominent in the CiOA model, early joint pain may predict OA outcomes in females only.

## Conclusions

It was clearly demonstrated that pain levels correlate to OA changes in females, whereas in males this relationship is nearly absent. This corroborates with the hypothesis that sex is a determinant in the link between pain and OA damage. Reproducibility was tested on CiOA gait analysis database experiments. Additionally, a recent clinical cohort study indicated that this phenomenon is also present clinically (Sellam et al., 2021). To our knowledge, this clinical study was the first to perform a similar, disaggregated analysis per sex concerning the pain-pathology connection. Our data underpin that pain pathways may be different between males and females in experimentally-induced OA and warrants further, more detailed analysis of males *versus* females in future OA experiments in general, but those on pain, in particular.

The identification of key factors that may be different between the sexes, using sex as a determining point, may lead to a more effective OA pain management. Moreover, this knowledge may contribute to the development of an advanced patient-oriented OA therapy, and the use of pain as an outcome measure in intervention studies may be treated as sex-pathway dependent.

##  Supplemental Information

10.7717/peerj.15482/supp-1 Supplemental Information 1 Supplemental methods Criteria for humane endpoint

10.7717/peerj.15482/supp-2 Table S1 Heat map correlation values Heatmap based on the Pearson coefficient values (r) between OA histological parameters and the different AUC pain parameters during the (A) whole time course, (B) early phase and (C) late phase. Coefficient values are displayed for significant correlations followed by the level of significance (*). Pearson coefficient values range from +1 and −1, with +1 as a perfect positive correlation, 0 as no correlation and −1 as a perfect negative correlation. * *p* < 0.05 and ** *p* < 0.01. d. Cannot be computed because at least one of the variables is constant.

10.7717/peerj.15482/supp-3 Table S2 Heat map correlation values combining all data from the historical dataset as well as the experiments Heatmap based on the Pearson coefficient values (r) between OA histological parameters and the different AUC pain parameters during the (A) whole time course, (B) early phase and (C) late phase. Coefficient values are displayed for significant correlations followed by the level of significance (*). Pearson coefficient values range from +1 and −1, with +1 as a perfect positive correlation, 0 as no correlation and −1 as a perfect negative correlation. * *p* < 0.05 and ** *p* < 0.01.

10.7717/peerj.15482/supp-4 Figure S1 Gait parameters measured in the contralateral and ipsilateral hinds Gait was evaluated using the Catwalk system. For all parameters, values are corrected for individual baseline. (A) LH/RH Print area. (B) LH/RH Max contact max intensity. Two-way ANOVA RM and Bonferroni’s multiple comparisons test. * (black asterisk) male vs female comparison; @ female to baseline and # male to baseline comparison. * *p* < 0.05, ** *p* < 0.01 and *** *p* < 0.001. N=10 mice per group. Data are expressed as means ± SD. LH: Left hind; RH: Right hind.

10.7717/peerj.15482/supp-5 Figure S2 Females with more cartilage damage and joint dislocation display more pain-related behavior than males Bubble plot shows the association between multiple parameters: cartilage damage, pain-related behavior, joint dislocations and sex. Pain-related behavior are displayed in the y-axis: (A) Duty cycle and (B) Single stance AUC during the early phase, cartilage damage in the x-axis, females are black and males white circles and animals with joint dislocation are depicted with bigger circles. Males: *n* = 20 and Females: *n* = 30

10.7717/peerj.15482/supp-6 Figure S3 When data analysis is performed aggregating males and females together, level of pain shows moderate correlation to OA pathology Heatmap based on the Pearson coefficient values (r) between OA histological parameters and the different AUC pain parameters during the whole time course (top), early phase (middle) and late phase (bottom). Coefficient values are displayed for significant correlations followed by the level of significance (*). For non-significant correlations, the r values were omitted. All coefficient values were colour coded as blue for positive correlations, yellow for r close to zero and orange for negative correlations. Pearson coefficient values range from +1 and −1, with +1 as a perfect positive correlation, 0 as no correlation and −1 as a perfect negative correlation. (N=20) * *p* < 0.01.

10.7717/peerj.15482/supp-7 Data S1 Raw data

10.7717/peerj.15482/supp-8 Supplemental Information 8 ARRIVE 2.0 Checklist

## Additional Information and Declarations

### Data Availability

The following information was supplied regarding data availability: The raw data is available in the Supplemental Files.
