# Supplementary material for: Early pain in females is linked to late pathological features in murine experimental osteoarthritis
Source: PeerJ. 2023 Jun 22;11:e15482. doi: 10.7717/peerj.15482 (PMC10290834; doi:10.7717/peerj.15482)
Supplement: Supplemental Information 1 — Criteria for humane endpoint [file peerj-11-15482-s001.docx]

Criteria for humane endpoint were set prior the experiments, as followed:

a) decreased movement through the cages with incapability of reaching food or water after having placed food on the bottom of the cage and placing bottles with longer nozzles;

b) self mutilation, characterized by damage to their feet or toes and

c) weight loss of 15 % in 2 days or 20% overall.

Intentionally, the length of the experiments was chosen so that animals were unlikely to reach these criteria.
