# Supplementary material for: Early pain in females is linked to late pathological features in murine experimental osteoarthritis
Source: PeerJ. 2023 Jun 22;11:e15482. doi: 10.7717/peerj.15482 (PMC10290834; doi:10.7717/peerj.15482)
Supplement: Table S1 — Heatmap based on the Pearson coefficient values (r) between OA histological parameters and the different AUC pain parameters during the (A) whole time course, (B) early phase and (C) late phase. Coefficient values are displayed for significant correlations followed by the level of significance (*). Pearson coefficient values range from +1 and −1, with +1 as a perfect positive correlation, 0 as no correlation and −1 as a perfect negative correlation. * p < 0.05 and ** p < 0.01. d. Cannot be computed because at least one of the variables is constant. [file peerj-11-15482-s002.pdf]

A)

	Female						Male					
	Cartilage damage	Dislocation	Osteophyte number	Synovial thickness	Synovial cellularity	**Total Time course**	Cartilage damage	Dislocation	Osteophyte number	Synovial thickness	Synovial cellularity	
Pearson Correlation	,922**	,700*	0,306	0,149	0,453		0,376	0,356	0,485	0,114	0,504	Pearson Correlation
Sig. (2-tailed)	0,000	0,024	0,390	0,681	0,189	**Incapacitance**	0,285	0,313	0,155	0,754	0,137	Sig. (2-tailed)
N	10	10	10	10	10		10	10	10	10	10	N
Pearson Correlation	,702*	,934**	0,543	0,551	0,255		0,015	-0,249	0,225	0,001	0,256	Pearson Correlation
Sig. (2-tailed)	0,024	0,000	0,104	0,099	0,478	**PAM**	0,967	0,488	0,533	0,998	0,474	Sig. (2-tailed)
N	10	10	10	10	10		10	10	10	10	10	N
Pearson Correlation	0,612	0,078	0,334	0,383	-0,120		,748*	0,631	,645*	0,502	0,588	Pearson Correlation
Sig. (2-tailed)	0,060	0,830	0,346	0,275	0,741	**Max Contact Max Intensity**	0,013	0,051	0,044	0,139	0,074	Sig. (2-tailed)
N	10	10	10	10	10		10	10	10	10	10	N
Pearson Correlation	,662*	,774**	0,579	0,391	0,482		0,376	0,417	0,553	0,528	0,328	Pearson Correlation
Sig. (2-tailed)	0,037	0,009	0,079	0,264	0,158	**Print Area**	0,284	0,231	0,097	0,117	0,355	Sig. (2-tailed)
N	10	10	10	10	10		10	10	10	10	10	N
Pearson Correlation	,906**	0,582	,665*	0,553	0,378		0,194	0,270	0,151	0,271	0,375	Pearson Correlation
Sig. (2-tailed)	0,000	0,078	0,036	0,097	0,282	**Duty Cycle**	0,590	0,451	0,678	0,449	0,285	Sig. (2-tailed)
N	10	10	10	10	10		10	10	10	10	10	N
Pearson Correlation	,794**	0,433	,680*	0,531	0,434		-0,035	-0,071	-0,085	-0,048	0,055	Pearson Correlation
Sig. (2-tailed)	0,006	0,211	0,030	0,115	0,210	**Stand time**	0,923	0,846	0,815	0,896	0,880	Sig. (2-tailed)
N	10	10	10	10	10		10	10	10	10	10	N
Pearson Correlation	,798**	,781**	,642*	0,474	0,463		0,545	0,555	0,475	,676*	0,604	Pearson Correlation
Sig. (2-tailed)	0,006	0,008	0,045	0,166	0,177	**Swing time**	0,103	0,096	0,166	0,032	0,065	Sig. (2-tailed)
N	10	10	10	10	10		10	10	10	10	10	N
Pearson Correlation	0,099	-0,155	0,341	0,164	0,303		-0,044	-0,111	0,466	0,533	0,182	Pearson Correlation
Sig. (2-tailed)	0,786	0,668	0,335	0,650	0,395	**Single Stance**	0,904	0,761	0,174	0,113	0,615	Sig. (2-tailed)
N	10	10	10	10	10		10	10	10	10	10	N
Pearson Correlation	,815**	,934**	,658*	,710*	0,372		0,393	0,358	0,238	0,422	0,502	Pearson Correlation
Sig. (2-tailed)	0,004	0,000	0,038	0,021	0,290	**Initial Dual Stance**	0,261	0,310	0,508	0,225	0,139	Sig. (2-tailed)
N	10	10	10	10	10		10	10	10	10	10	N
Pearson Correlation	-0,302	-0,373	-0,128	-0,281	-0,548		0,299	0,253	0,376	0,260	0,191	Pearson Correlation
Sig. (2-tailed)	0,397	0,288	0,724	0,432	0,101	**Terminal Dual Stance**	0,401	0,480	0,284	0,468	0,597	Sig. (2-tailed)
N	10	10	10	10	10		10	10	10	10	10	N

B)

	Female						Male					
	Cartilage damage	Dislocation	Osteophyte number	Synovial thickness	Synovial cellularity	Early phase	Cartilage damage	Dislocation	Osteophyte number	Synovial thickness	Synovial cellularity	
Pearson Correlation	,716*	,635*	0,402	-0,118	0,533	**Incapacitance Early**	0,383	0,376	0,488	0,068	0,513	Pearson Correlation
Sig. (2-tailed)	0,020	0,048	0,249	0,745	0,113		0,274	0,285	0,153	0,853	0,129	Sig. (2-tailed)
N	10	10	10	10	10		10	10	10	10	10	N
Pearson Correlation	0,616	,850**	0,579	0,510	0,301	**PAM Early**	0,128	-0,006	0,063	-0,132	0,031	Pearson Correlation
Sig. (2-tailed)	0,058	0,002	0,079	0,132	0,398		0,724	0,987	0,862	0,716	0,932	Sig. (2-tailed)
N	10	10	10	10	10		10	10	10	10	10	N
Pearson Correlation	,700*	0,149	0,420	0,456	-0,035	**Max Contact Max Intensity Early**	0,630	0,594	0,528	0,602	0,540	Pearson Correlation
Sig. (2-tailed)	0,024	0,681	0,227	0,186	0,925		0,051	0,070	0,116	0,066	0,107	Sig. (2-tailed)
N	10	10	10	10	10		10	10	10	10	10	N
Pearson Correlation	,672*	,777**	0,573	0,370	0,472	**Print Area Early**	0,479	0,581	0,618	0,626	0,422	Pearson Correlation
Sig. (2-tailed)	0,033	0,008	0,084	0,292	0,168		0,162	0,078	0,057	0,053	0,224	Sig. (2-tailed)
N	10	10	10	10	10		10	10	10	10	10	N
Pearson Correlation	,882**	0,602	,677*	0,572	0,396	**Duty Cycle Early**	0,462	0,566	0,386	0,567	0,619	Pearson Correlation
Sig. (2-tailed)	0,001	0,066	0,031	0,084	0,257		0,179	0,088	0,270	0,088	0,057	Sig. (2-tailed)
N	10	10	10	10	10		10	10	10	10	10	N
Pearson Correlation	,756*	0,393	,652*	0,550	0,442	**Stand time Early**	0,098	0,078	-0,019	0,013	0,229	Pearson Correlation
Sig. (2-tailed)	0,011	0,262	0,041	0,100	0,201		0,789	0,831	0,959	0,972	0,524	Sig. (2-tailed)
N	10	10	10	10	10		10	10	10	10	10	N
Pearson Correlation	,828**	,792**	0,627	0,507	0,514	**Swing time Early**	0,559	0,548	0,495	,688*	0,589	Pearson Correlation
Sig. (2-tailed)	0,003	0,006	0,052	0,135	0,128		0,093	0,101	0,145	0,028	0,073	Sig. (2-tailed)
N	10	10	10	10	10		10	10	10	10	10	N
Pearson Correlation	0,130	0,088	0,448	0,265	0,299	**Single Stance Early**	0,155	0,181	0,612	,735*	0,343	Pearson Correlation
Sig. (2-tailed)	0,721	0,808	0,194	0,459	0,401		0,669	0,616	0,060	0,015	0,332	Sig. (2-tailed)
N	10	10	10	10	10		10	10	10	10	10	N
Pearson Correlation	,720*	,798**	0,604	,717*	0,363	**Initial Dual Stance Early**	0,416	0,353	0,254	0,435	0,514	Pearson Correlation
Sig. (2-tailed)	0,019	0,006	0,064	0,020	0,302		0,232	0,318	0,480	0,209	0,128	Sig. (2-tailed)
N	10	10	10	10	10		10	10	10	10	10	N
Pearson Correlation	-0,132	-0,252	0,123	0,068	-0,143	**Terminal Dual Stance Early**	0,245	0,279	0,614	0,568	0,209	Pearson Correlation
Sig. (2-tailed)	0,717	0,482	0,736	0,851	0,693		0,495	0,435	0,059	0,087	0,563	Sig. (2-tailed)
N	10	10	10	10	10		10	10	10	10	10	N

C)

	Female						Male					
	Cartilage damage	Dislocation	Osteophyte number	Synovial thickness	Synovial cellularity	Late phase	Cartilage damage	Dislocation	Osteophyte number	Synovial thickness	Synovial cellularity	
Pearson Correlation	,952**	0,600	0,109	0,393	0,175	**Incapacitance Late**	0,013	-0,271	0,007	,769**	0,014	Pearson Correlation
Sig. (2-tailed)	0,000	0,067	0,764	0,262	0,628		0,971	0,449	0,984	0,009	0,970	Sig. (2-tailed)
N	10	10	10	10	10		10	10	10	10	10	N
Pearson Correlation	,731*	,635*	0,234	0,378	-0,039	**PAM Late**	-0,118	-0,415	0,203	0,017	0,234	Pearson Correlation
Sig. (2-tailed)	0,016	0,049	0,514	0,281	0,915		0,745	0,233	0,574	0,963	0,515	Sig. (2-tailed)
N	10	10	10	10	10		10	10	10	10	10	N
Pearson Correlation	0,443	-0,039	0,182	0,250	-0,247	**Max Contact Max Intensity Late**	0,195	0,033	0,156	-0,246	0,018	Pearson Correlation
Sig. (2-tailed)	0,200	0,915	0,614	0,486	0,491		0,589	0,928	0,668	0,493	0,961	Sig. (2-tailed)
N	10	10	10	10	10		10	10	10	10	10	N
Pearson Correlation	.[d]	.[d]	.[d]	.[d]	.[d]	**Print Area Late**	-0,313	-0,475	-0,267	-0,364	-0,358	Pearson Correlation
Sig. (2-tailed)							0,379	0,166	0,456	0,301	0,310	Sig. (2-tailed)
N	10	10	10	10	10		10	10	10	10	10	N
Pearson Correlation	,829**	0,368	0,598	0,505	0,314	**Duty Cycle Late**	-0,039	0,019	-0,055	-0,012	0,145	Pearson Correlation
Sig. (2-tailed)	0,003	0,295	0,068	0,136	0,377		0,915	0,959	0,879	0,973	0,689	Sig. (2-tailed)
N	10	10	10	10	10		10	10	10	10	10	N
Pearson Correlation	,759*	0,376	,647*	0,5212	0,348	**Stand time Late**	-0,106	-0,145	-0,127	-0,106	-0,023	Pearson Correlation
Sig. (2-tailed)	0,011	0,285	0,043	0,122	0,324		0,771	0,690	0,726	0,771	0,951	Sig. (2-tailed)
N	10	10	10	10	10		10	10	10	10	10	N
Pearson Correlation	0,312	0,340	0,485	0,346	0,259	**Swing time Late**	-0,534	-0,569	-,680*	-0,540	-,692*	Pearson Correlation
Sig. (2-tailed)	0,380	0,336	0,156	0,328	0,469		0,112	0,086	0,030	0,107	0,027	Sig. (2-tailed)
N	10	10	10	10	10		10	10	10	10	10	N
Pearson Correlation	0,176	-0,393	0,084	0,011	0,193	**Single Stance Late**	-0,341	-0,537	0,069	0,024	-0,097	Pearson Correlation
Sig. (2-tailed)	0,627	0,261	0,819	0,976	0,594		0,334	0,109	0,851	0,948	0,790	Sig. (2-tailed)
N	10	10	10	10	10		10	10	10	10	10	N
Pearson Correlation	,841**	,929**	,654*	,697*	0,382	**Initial Dual Stance Late**	0,393	0,357	0,239	0,419	0,497	Pearson Correlation
Sig. (2-tailed)	0,002	0,000	0,040	0,025	0,276		0,262	0,312	0,506	0,229	0,144	Sig. (2-tailed)
N	10	10	10	10	10		10	10	10	10	10	N
Pearson Correlation	-0,313	-0,256	-0,401	-0,578	-,695*	**Terminal Dual Stance Late**	0,019	-0,052	-0,254	-0,271	-0,125	Pearson Correlation
Sig. (2-tailed)	0,378	0,476	0,250	0,080	0,026		0,959	0,888	0,478	0,448	0,730	Sig. (2-tailed)
N	10	10	10	10	10		10	10	10	10	10	N
