# Supplementary material for: Early pain in females is linked to late pathological features in murine experimental osteoarthritis
Source: PeerJ. 2023 Jun 22;11:e15482. doi: 10.7717/peerj.15482 (PMC10290834; doi:10.7717/peerj.15482)
Supplement: Table S2 — Heatmap based on the Pearson coefficient values (r) between OA histological parameters and the different AUC pain parameters during the (A) whole time course, (B) early phase and (C) late phase. Coefficient values are displayed for significant correlations followed by the level of significance (*). Pearson coefficient values range from +1 and −1, with +1 as a perfect positive correlation, 0 as no correlation and −1 as a perfect negative correlation. * p < 0.05 and ** p < 0.01. [file peerj-11-15482-s003.pdf]

A)

	Female			Male		
	Cartilage damage	Dislocation	Total Time course	Cartilage damage	Dislocation	
Pearson Correlation	-0,005	0,096	**Max Contact Max Intensity**	0,315	0,363	Pearson Correlation
Sig. (2-tailed)	0,980	0,616		0,176	0,116	Sig. (2-tailed)
N	30	30		20	20	N
Pearson Correlation	0,124	0,219	**Print Area**	0,340	0,245	Pearson Correlation
Sig. (2-tailed)	0,514	0,244		0,142	0,297	Sig. (2-tailed)
N	30	30		20	20	N
Pearson Correlation	,376*	,436*	**Duty Cycle**	0,023674901	0,136	Pearson Correlation
Sig. (2-tailed)	0,041	0,016		0,921	0,567	Sig. (2-tailed)
N	30	30		20	20	N
Pearson Correlation	,551**	,559**	**Single Stance**	0,054	0,105	Pearson Correlation
Sig. (2-tailed)	0,002	0,001		0,820	0,658	Sig. (2-tailed)
N	30	30		20	20	N
Pearson Correlation	0,039	0,186	**Initial Dual Stance**	0,261	0,275	Pearson Correlation
Sig. (2-tailed)	0,838	0,324		0,266	0,241	Sig. (2-tailed)
N	30	30		20	20	N
Pearson Correlation	0,102	-0,138	**Terminal Dual Stance**	0,014	0,068	Pearson Correlation
Sig. (2-tailed)	0,590	0,466		0,952	0,777	Sig. (2-tailed)
N	30	30		20	20	N

B)

	Female			Male		
	Cartilage damage	Dislocation	Early phase	Cartilage damage	Dislocation	
Pearson Correlation	0,159	0,231	**Max Contact Max Intensity Early**	0,287	0,376	Pearson Correlation
Sig. (2-tailed)	0,401	0,219		0,219	0,103	Sig. (2-tailed)
N	30	30		20	20	N
Pearson Correlation	0,294	0,356	**Print Area Early**	0,313	0,343	Pearson Correlation
Sig. (2-tailed)	0,115	0,053		0,180	0,139	Sig. (2-tailed)
N	30	30		20	20	N
Pearson Correlation	,500**	,524**	**Duty Cycle Early**	0,123	0,233	Pearson Correlation
Sig. (2-tailed)	0,005	0,003		0,605	0,322	Sig. (2-tailed)
N	30	30		20	20	N
Pearson Correlation	,610**	,561**	**Single Stance Early**	0,115	0,233	Pearson Correlation
Sig. (2-tailed)	0,000	0,001		0,630	0,324	Sig. (2-tailed)
N	30	30		20	20	N
Pearson Correlation	0,005	0,086	**Initial Dual Stance Early**	0,280	0,282	Pearson Correlation
Sig. (2-tailed)	0,979	0,650		0,232	0,228	Sig. (2-tailed)
N	30	30		20	20	N
Pearson Correlation	0,184	-0,011	**Terminal Dual Stance Early**	0,039	0,157	Pearson Correlation
Sig. (2-tailed)	0,330	0,953		0,871	0,509	Sig. (2-tailed)
N	30	30		20	20	N

C)

	Female			Male		
	Cartilage damage	Dislocation	Late phase	Cartilage damage	Dislocation	
Pearson Correlation	-0,133	0,006	**Max Contact Max Intensity Late**	0,199	0,125	Pearson Correlation
Sig. (2-tailed)	0,483	0,977		0,399	0,599	Sig. (2-tailed)
N	30	30		20	20	N
Pearson Correlation	-0,183	-0,022	**Print Area Late**	0,163	-0,044	Pearson Correlation
Sig. (2-tailed)	0,334	0,909		0,493	0,853	Sig. (2-tailed)
N	30	30		20	20	N
Pearson Correlation	0,022	0,111	**Duty Cycle Late**	-0,080	0,027	Pearson Correlation
Sig. (2-tailed)	0,909	0,559		0,737	0,912	Sig. (2-tailed)
N	30	30		20	20	N
Pearson Correlation	0,032	0,136	**Single Stance Late**	0,037	-0,005	Pearson Correlation
Sig. (2-tailed)	0,866	0,473		0,876	0,985	Sig. (2-tailed)
N	30	30		20	20	N
Pearson Correlation	0,019	0,193	**Initial Dual Stance Late**	0,245	0,251	Pearson Correlation
Sig. (2-tailed)	0,921	0,306		0,299	0,286	Sig. (2-tailed)
N	30	30		20	20	N
Pearson Correlation	-0,105	-0,263	**Terminal Dual Stance Late**	-0,105	-0,137	Pearson Correlation
Sig. (2-tailed)	0,580	0,160		0,660	0,565	Sig. (2-tailed)
N	30	30		20	20	N
