# Supplementary figures and images for: Early pain in females is linked to late pathological features in murine experimental osteoarthritis
Source: PeerJ. 2023 Jun 22;11:e15482. doi: 10.7717/peerj.15482 (PMC10290834; doi:10.7717/peerj.15482)

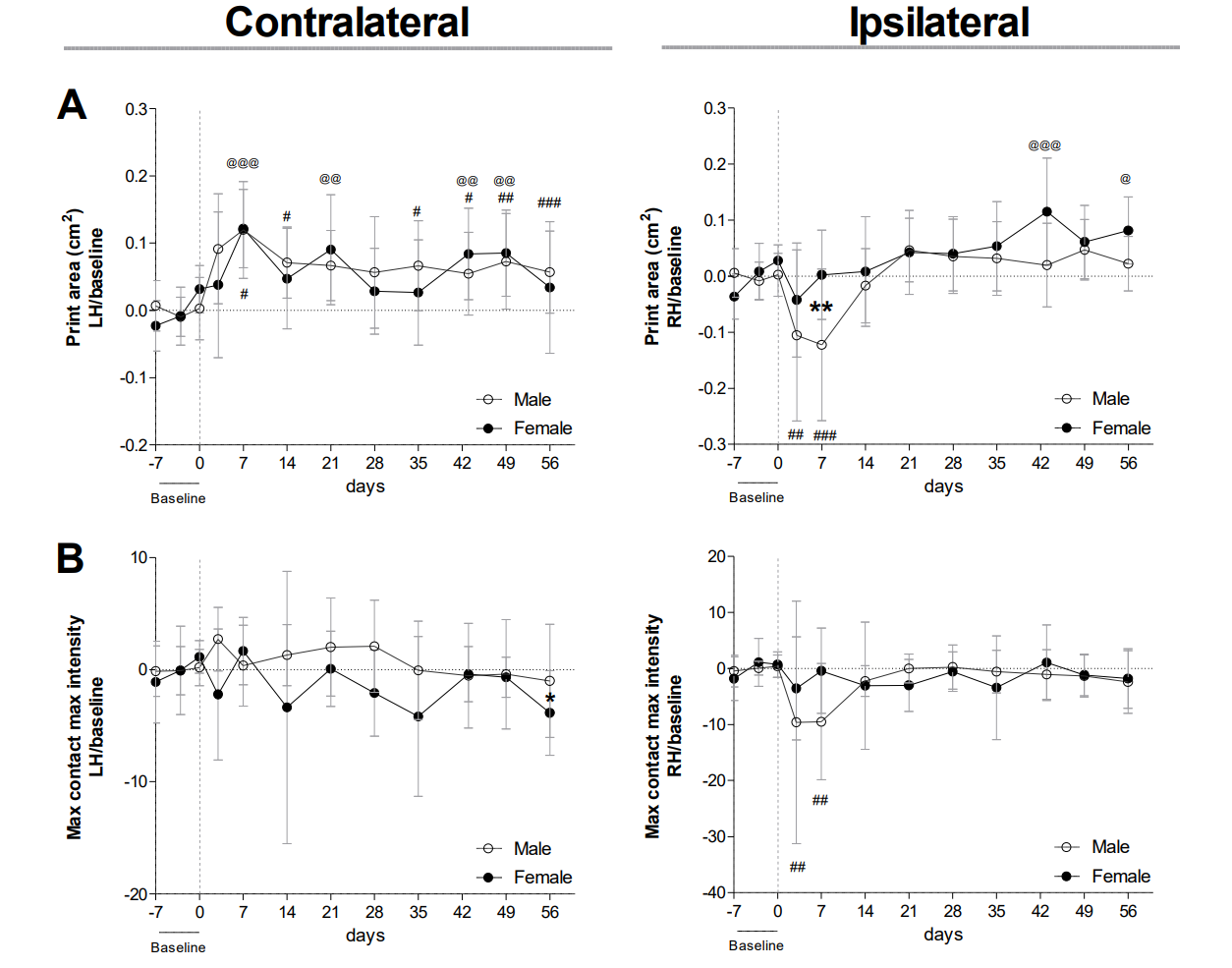

Supplement: Figure S1 — Gait was evaluated using the Catwalk system. For all parameters, values are corrected for individual baseline. (A) LH/RH Print area. (B) LH/RH Max contact max intensity. Two-way ANOVA RM and Bonferroni’s multiple comparisons test. * (black asterisk) male vs female comparison; @ female to baseline and # male to baseline comparison. * p < 0.05, ** p < 0.01 and *** p < 0.001. N=10 mice per group. Data are expressed as means ± SD. LH: Left hind; RH: Right hind. [file peerj-11-15482-s004.png]

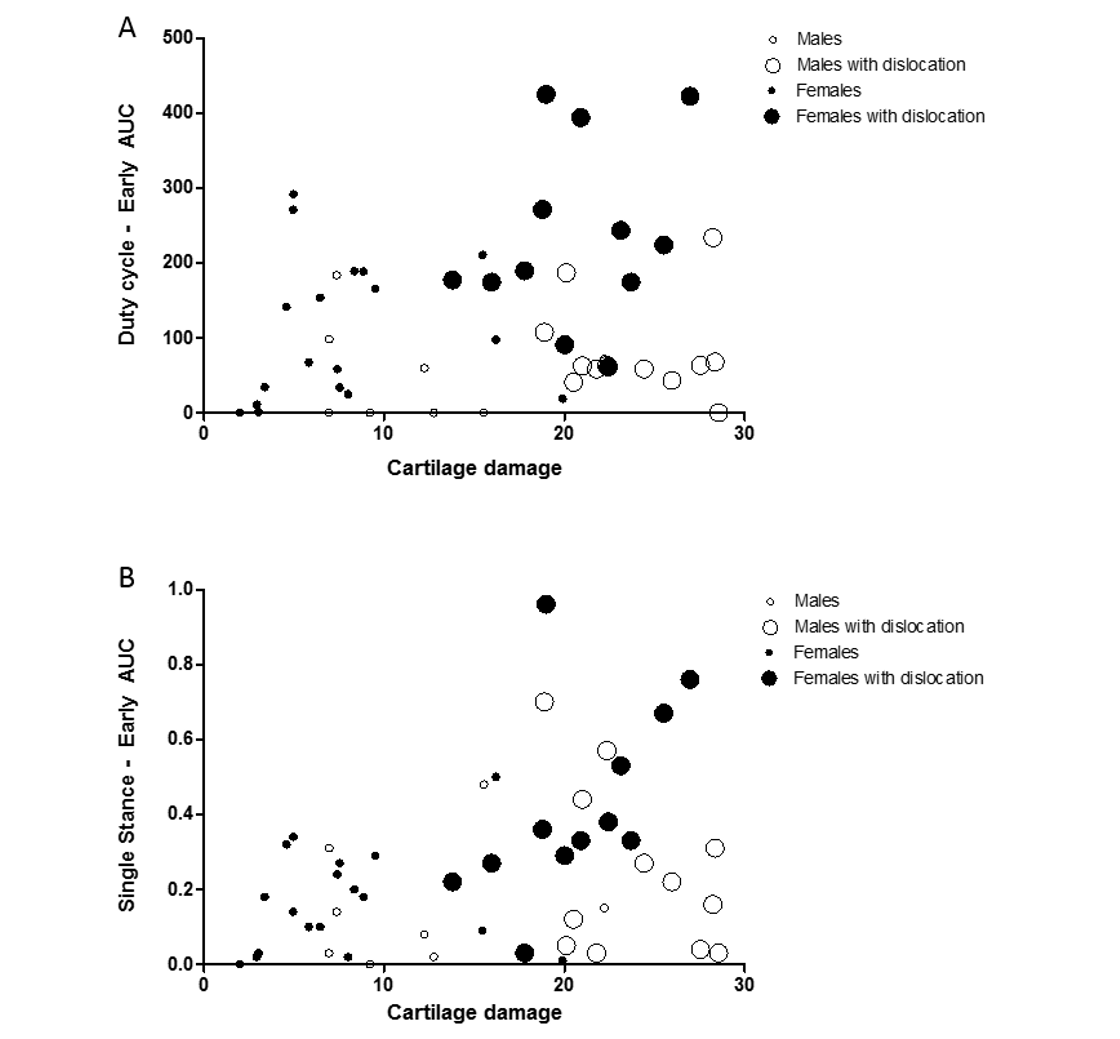

Supplement: Figure S2 — Bubble plot shows the association between multiple parameters: cartilage damage, pain-related behavior, joint dislocations and sex. Pain-related behavior are displayed in the y-axis: (A) Duty cycle and (B) Single stance AUC during the early phase, cartilage damage in the x-axis, females are black and males white circles and animals with joint dislocation are depicted with bigger circles. Males: n = 20 and Females: n = 30 [file peerj-11-15482-s005.png]

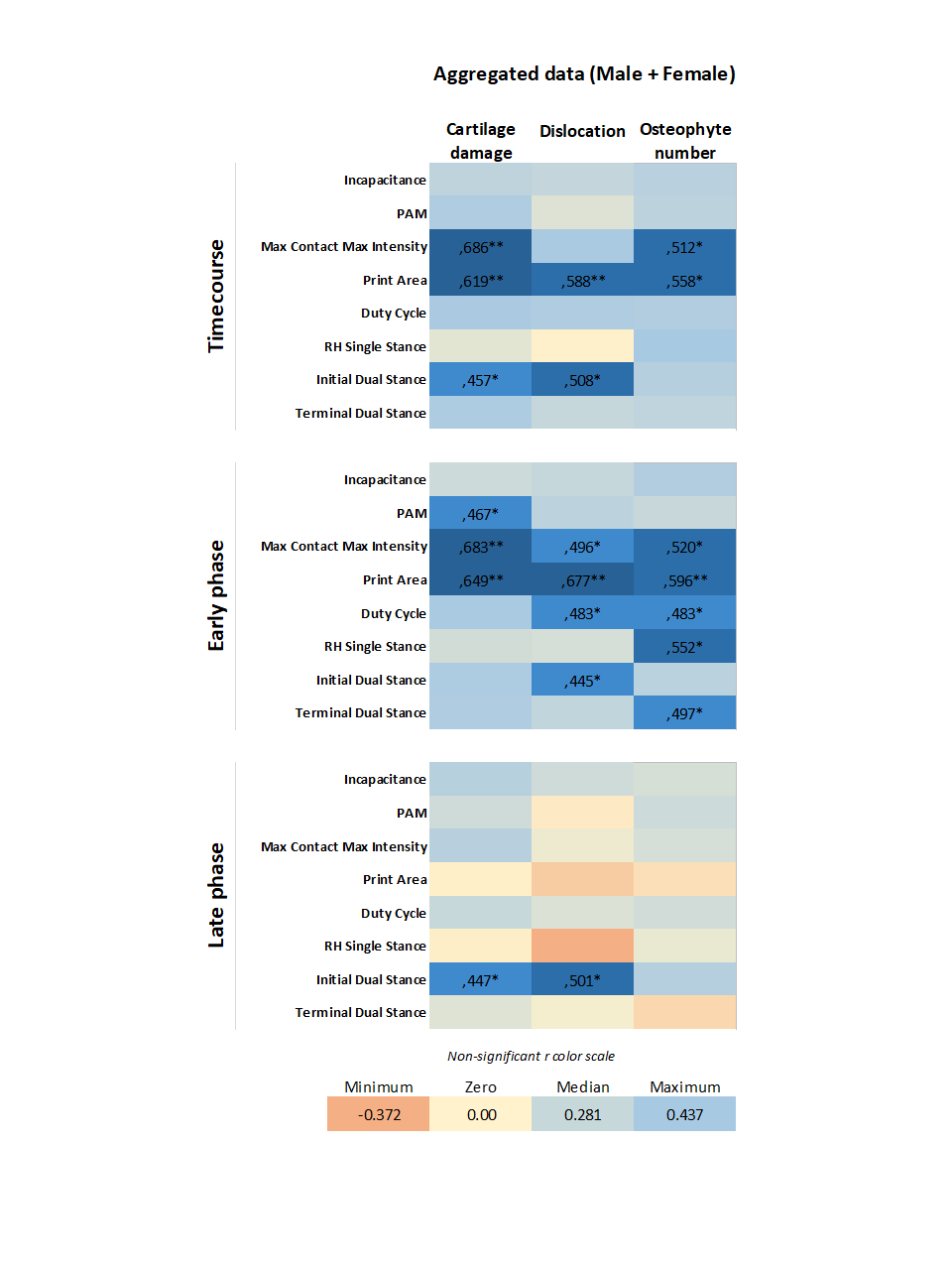

Supplement: Figure S3 — Heatmap based on the Pearson coefficient values (r) between OA histological parameters and the different AUC pain parameters during the whole time course (top), early phase (middle) and late phase (bottom). Coefficient values are displayed for significant correlations followed by the level of significance (*). For non-significant correlations, the r values were omitted. All coefficient values were colour coded as blue for positive correlations, yellow for r close to zero and orange for negative correlations. Pearson coefficient values range from +1 and −1, with +1 as a perfect positive correlation, 0 as no correlation and −1 as a perfect negative correlation. (N=20) * p < 0.01. [file peerj-11-15482-s006.png]
